# Inconsistency, Thou Art—A State Medical Journal

**Published:** 1907-11

**Authors:** 


### SELECTIONS AND ABSTRACTS.

### INCONSISTENCY, THOU ART—A STATE MEDICAL JOURNAL.

With all the vituperation and anathemas that have been poured out on the heads of proprietary pharmaceuticals, it is astounding how our brethren of the redeemed(?) organization press have the hardihood to continue to replenish their coffers with receipts from the advertising thereof. Some time ago the Journal of the Michigan State Medical Society published the following regulation:

Advertising.

The Council recommended the adoption of the following rules regarding advertising in the State journal:

Advertisements of remedies expressly disapproved by the Council of Pharmacy shall not be admitted.

The Journal does not stand sponsor for advertisements in its pages or necessarily approve of those published.

Reading notices of advertisements shall not be admitted afer contracts in existence have expired.

The July issue of the same publication also contains the following:

"The rules regulating the admission of advertisements, as established by the Council are: (1) Remedies approved by the Council on Pharmacy of the American Medical Association may be admitted. (2) Remedies disapproved by the Council on Pharmacy will not be admitted. (3) Remedies advertised to the laity will not be admitted."

And yet, mirabile dictu, a perusal of the advertising pages of the latest issue of this sanctimonious publication shows that eighty per cent. of its income is derived from the advertisements of products that have either been expressly disapproved, or that certainly have not been approved, by the Council on Pharmacy and Chemistry!

What is an honest man to decide from such a condition? That a Society does not control its organ, or that the ethical(?) publishing situation is as rotten as the abuses it pretends to correct?

Editor(?) Philip Mills Jones, of the California State Journal of Medicine, has placed a two-year time limit on the defenceless advertisers in the publication he adorns, and then—quick curtain! Meanwhile, Editor(?) Jones neglects to state how useful the receipts from such "probationary" advertisers will be during this two-year period, or to what extent they will help him to draw his salary with ethical regularity. Au contraire, Editor(?) Jones, like Senator Sorghum, has a convenient habit of ignoring details that would explain his attitude on many questions. He believes that to make his views on such questions absolutely clear, or honestly explicit, would place his readers and others in a position to pin him down to them on occasions when policy dictates the reverse, and so the world is left to draw its own conclusions.

Considered without sophistry, however, the whole question of advertising censorship is one that shows little else than gross inconsistencies, personal antagonisms and a deliberately and well-planned attempt to discredit therapeutics generally. As long as medical opinions are shaped and promulgated by men who have made no greater contributions to medical science than the present mouthpieces of the organized profession, this unfortunate condition is bound to continue.

Some day the advertising atmosphere will be cleared, abuses will be recognized and properly overcome, and the good and worthy features of the pharmaceutical industry, with renewed vigor, will go on contributing to honest, hopeful medicine.

But it will not be "Sans-culottes" M. Jones, George H. Talleyrand Simmons, or one or another of their rough-riding, inconsistent and often-times unscrupulous co-adjutors, that will do it.—American Medical Journalist.

---

Texas Medical News.—Editor M. M. Smith has moved, with his journal and family, from Austin to Dallas, Tex.

